# The Association of Serum Neurofilament Light Chain and Acute Ischaemic Stroke Is Influenced by Effective Revascularization

**DOI:** 10.1155/2022/5236080

**Published:** 2022-05-03

**Authors:** Fa-Ying Zhou, Dong-Wan Chen, Hui-Yun Li, Chi Zhu, Ying-Ying Shen, Ze-Yan Peng, Ling Li, Xian-Le Bu, Gui-Hua Zeng, Meng Zhang, Yan-Jiang Wang, Wang-Sheng Jin

**Affiliations:** Department of Neurology and Centre for Clinical Neuroscience, Daping Hospital, Third Military Medical University, 10 Changjiang Branch Road, Yuzhong District, Chongqing 400042, China

## Abstract

**Objective:**

To explore associations of serum neurofilament light chain (sNfL) at admission with clinical deficits and the long-term prognosis of acute ischaemic stroke (AIS).

**Methods:**

We recruited 110 AIS patients with serum sampled at hospital arrival. The concentrations of sNfL were detected by a Simoa HD-1 analyser. We first investigated the determinants of sNfL levels at admission within the study population. Associations of sNfL levels with National Institutes of Health Stroke Scale (NIHSS) scores and modified Rankin Scale (mRS) scores were then tested. We further divided the patients into revascularized and nonrevascularized groups, and the associations of sNfL levels with NIHSS and mRS scores were assessed in these subgroups.

**Results:**

Age, sex, stroke history, and the time between the onset of illness and arrival at the hospital were independent influencing factors of sNfL levels within the study population. The sNfL levels at admission were correlated with the NIHSS scores 7 days after stroke (*p* = 0.004) across all subjects but showed no correlation with the NIHSS scores at admission (*p* = 0.293) or the mRS scores 6 months after stroke (*p* = 0.065). Further analysis revealed that in the nonrevascularized group of AIS patients, the sNfL levels at admission were positively correlated with NIHSS scores (NIHSS at admission, *p* = 0.005; NIHSS 7 days after stroke, *p* = 0.003) and negatively correlated with mRS scores (*p* = 0.011).

**Conclusion:**

sNfL levels at admission could be a potential biomarker for predicting clinical deficits and prognosis in the natural course of AIS.

## 1. Introduction

Acute ischaemic stroke (AIS) is a major cause of mortality and morbidity worldwide and imposes a huge burden on society [1, 2]. “Time is brain”, rapid diagnosis, and disease assessment are key factors in the treatment of AIS [3, 4]. Imaging technology has developed rapidly, and computed tomography (CT) and magnetic resonance imaging (MRI) can assess the responsible vessels and infarct location in the early stages of AIS. However, the assessment of the extent of ischaemia in the brain is based on the changes in the magnetic resonance signal of brain tissue after ischaemia and hypoxia, which does not directly represent the damage and death of neural cells. Even in some cases, there is negative imaging in the early stage of AIS [5]. Therefore, the study of sensitive and reliable peripheral biomarkers is of great significance.

Neurofilament light chain (NfL) is one of the most important structural proteins in the neuronal cytoskeleton. It is released into the blood when the axons of neurons and glial cells are damaged. Therefore, serum NfL (sNfL) is elevated in neurodegenerative diseases [6–8], neuroimmune diseases [9, 10], and stroke-related lesions [11]. Several studies have found that sNfL levels can increase rapidly in the early stages of AIS and correlate with the severity of disease [12, 13].

Most previous studies examined correlations between sNfL levels and the severity and prognosis of stroke at a fixed time point after stroke. However, most patients do not arrive at the hospital and have their serum sampled at a consistent time. Sampling at admission is closer to actual clinical practice, and therefore, there is the question of whether the sNfL level at admission can be used as an effective marker for AIS patients. With advancements in technology, timely and effective revascularization can change the natural course of stroke. Can sNfL levels be a reliable predictive biomarker for patients who successfully undergo revascularization? Considering this question, our study was conducted to investigate the correlation of sNfL levels at admission with neurological deficits and the prognosis of AIS. Furthermore, we investigated the effect of revascularization on the correlation between sNfL levels at admission and AIS.

## 2. Methods

### 2.1. Study Population

**Figure 1:**
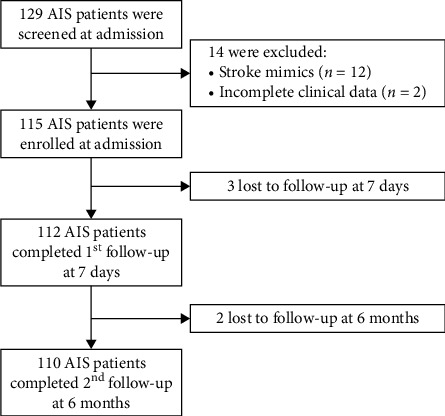
Flowchart illustrating patient enrolment/follow-up and available serum samples for serum neurofilament light chain (NfL) measurements.

We recruited 129 AIS patients from Chongqing Daping Hospital from 2017 to 2018. The inclusion criteria were as follows: (1) patients with a diagnosis of AIS within 7 days of disease onset, (2) patients with an NIHSS score between 3 and 25 on admission to the hospital, and (3) patients and families who agreed to participate and signed informed consent forms. The exclusion criteria included (1) infarct patients with serious disturbances of consciousness and aphasia that prevented proper communication, (2) patients with a history of neurological disorders and those with sequelae or other serious physical illnesses that prevented them from submitting to inspections, (3) infarct patients with serious combined complications (such as between the heart and lung problems or liver problems and kidney failure), (4) patients with advanced cancer, and (5) patients with missing clinical information or those lost to follow-up. A CONSORT (Consolidated Standards of Reporting Trials) diagram of patients is depicted in Figure 1. Nineteen patients were lost in this process due to stroke mimics, death, or serious diseases of other causes. In total, 110 AIS patients were recruited for further analysis.

### 2.2. Clinical Assessment

The clinical assessment and diagnosis of AIS were performed by skilled clinical neurologists following the criteria provided by the World Health Organization based on patient history, clinical data, and neuroimaging results (computed tomography [CT] or magnetic resonance imaging [MRI]). Demographic data, including age, sex, cigarette smoking, alcohol intake, heart diseases (myocardial infarction, atrial fibrillation, and heart failure), hypertension, diabetes mellitus, medical history, and the time between the onset of illness and arrival at the hospital (arrival time), of all participants were thoroughly collected. The NIHSS and mRS scores were used to assess neurological function, clinical deficits, and outcomes of AIS at admission and 6 months poststroke, and both tests were performed by experienced neurologists.

### 2.3. Neuroimaging

Carotid and cranial arteries and cerebral hypoperfusion were illustrated by computed tomography angiography and perfusion (CTA+CTP). CTP was performed using a Philips Brilliance 64 CT scanner with the following parameters: collimator 128 ING, 80 kVp, and 90 mAs with total coverage of 40 mm. The plane of imaging was parallel to the floor of the anterior cranial fossa just above the orbits. A total of 288 images were obtained in a total of 18 cycles with a scan time of 46 seconds. CTP images were analysed using an IntelliSpace Portal station (Philips) and VPCT perfusion software, which was automatically performed based on prevalues of cerebral blood flow (CBF) and CBV. Nonviable and penumbra tissues were automatically determined depending on the prevalues of cerebral blood flow (CBF) and CBV. The baseline infarct core volume (mL) was initially calculated based on the CTP images using a CBV threshold of 2 mL/100 mL volume and then retrospectively using a CBV of 1.2 mL/100 mL. All AIS patients were confirmed to have the diagnosis based on the MRI-DWI results. DWI was performed using a 1.5 T MRI scanner (General Electric Medical Systems, Milwaukee, WI). The duration of the DWI pulse sequence was set to 2 minutes and 35 seconds. Images were automatically postprocessed to produce isotropic DW images and apparent diffusion coefficient maps.

### 2.4. Treatment

The patients were assessed by members of the stroke team, who determined the treatment strategies. Intravenous thrombolysis is recommended for patients who arrive within 4.5 hours after stroke, and intra-arterial thrombectomy is recommended for patients with carotid or cranial artery occlusions and ischaemic penumbra retention [14]. The other patients received a standardized therapy following secondary stroke prevention [15].

### 2.5. Sample Collection and Test

The blood was sampled and sent for haematologic examination upon patient arrival at the hospital. Blood samples were immediately collected upon hospital arrival in the emergency department. They were separated and stored within 2 hours after sampling at −80°C for future analysis. The concentrations of sNfL were measured using the commercially available single-molecule array (SIMOA) Human Neurology 3-Plex A assay kit on the automated SIMOA HD-1 analyser (Quanterix, Lexington, Massachusetts). The parameters of SIMOA were set as follows: the limit of detection (LOD) was set to 0.038 pg/mL (range: 0.003–0.079 pg/mL), and the lower limit of quantification (LLOQ) was set to 0.174 pg/mL (pooled CV: 15.3%; mean recovery: 105%).

### 2.6. Follow-Up

The follow-up of the AIS patients started at the time of diagnosis. An individualized approach was provided to the patients through outpatient office visits every month. The National Institutes of Health Stroke Scale (NIHSS) was evaluated at admission and 7 days after stroke, and modified Rankin Scale (mRS) scores were evaluated 6 months after stroke.

### 2.7. Statistical Analysis

SPSS (IBM Inc., Armonk, NY, version 19.0) statistical software was used. Discrete variables are expressed as counts (percentages), and continuous variables are expressed as medians (interquartile range [IQR]). sNfL was logarithmically (base e) transformed to have a normal distribution. To compare variables, we used Fisher's exact test (for categorical data) and the Mann–Whitney *U* test (for unmatched continuous data). For regression analyses, ln(sNfL) was treated as the dependent variable. The independent variables were age, sex, disease history, cholesterol, ASPECT scores, and time between the onset of illness and arrival at the hospital. Variables that had significant univariate associations (*p* < 0.05) with ln(sNfL) were included in a multivariable model. We applied linear regression analysis to assess associations of ln(sNfL) levels with NIHSS scores. Where indicated, adjustment was performed using analysis of residuals after regressing out confounders. Bivariate regression models were used to explore the association between ln(sNfL) levels and the outcome of stroke. We report odds ratios (ORs) along with 95% confidence intervals as measures of association and uncertainty, respectively. Testing was two-sided, and *p* values < 0.05 were considered to indicate statistical significance.

## 3. Results

### 3.1. Baseline Characteristics

The baseline characteristics of the study samples are presented in Table 1. Certain differences in the demographic and clinical characteristics were found between subgroups divided by treatment strategies. The patients who received successful intra-arterial thrombectomy without serious complications or thrombolysis and achieved better effects (i.e., their NIHSS scores rapidly decreased) were incorporated into the revascularized group. The remaining patients were incorporated into the nonrevascularized group, including those who received no thrombolysis or arterial thrombectomy, those who received one of these interventions but did not show better effects, and those with other complications. The revascularized group was more likely to suffer from arterial occlusion (*p* < 0.001), have higher NIHSS scores on admission (*p* = 0.005), and have shorter arrival times (*p* < 0.001) and lower ASPECT scores (*p* = 0.001) than the nonrevascularized group (Table 1). Meanwhile, the nonrevascularized group showed higher ln(sNfL) (*p* = 0.014) at admission than the revascularized group.

### 3.2. Correlations of sNfL Levels with Vascular Risk Factors and ASPECT Scores

**Figure 2:**
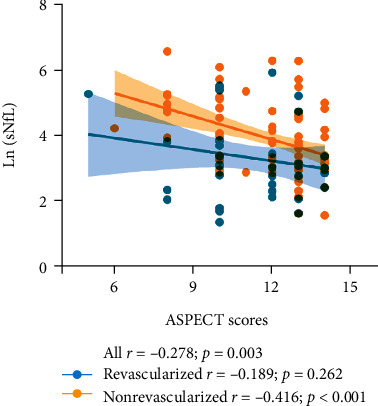
Associations between the ln(sNfL) and ASPECT scores in all patients: the revascularized group and the nonrevascularized group.

Age, sex, disease history, and arrival time were found to be significantly correlated with ln(sNfL) in both univariable and multivariable analyses (Table 2). Alberta Stroke Program Early CT Score (ASPECTS) scores are a widely used measure of ischaemic changes on noncontrast CT and were also significantly correlated with the ln(sNfL) in all patients (*r* = −0.278, *p* = 0.003) and the nonrevascularized group (*r* = −0.416, *p* < 0.001) (Figure 2).

### 3.3. Correlations of sNfL Levels with Clinical Deficits (NIHSS Scores)

In the present study, we explored the associations between ln(sNfL) at admission and NIHSS scores. The difference in NIHSS scores between admission and 7 days poststroke was considered an indicator of early recovery of neurological deficits. In all subjects, significant associations were found between ln(sNfL) at admission and both NIHSS scores at 7 days and the differences in NIHSS score in the univariable analysis, which remained significant after adjusting for age, sex, stroke history, and arrival time (Table 3). In the revascularized group, only the NIHSS score at admission was significantly correlated with ln(sNfL) at admission in the univariable analysis. ln(sNfL) was significantly correlated with NIHSS scores at both time points in the nonrevascularized group, and this correlation remained significant after adjusting for age, sex, stroke history, and arrival time (Table 3).

### 3.4. sNfL Levels Predict the Clinical Prognosis of AIS

We further explored the predictive value of sNfL levels at admission for the clinical prognosis of AIS. mRS scores at 6 months poststroke ranged from 0 to 6. The patients who achieved mRS scores of 0-2 at 6 months after stroke were included in the good prognosis group, while those who achieved mRS scores of 3-6 were included in the poor prognosis group. According to the results of simple logistic regression analyses, ln(sNfL) at admission was associated with mRS scores 6 months after stroke in the nonrevascularized group (Table 4). Across all subjects and in the revascularized group, no significant associations were found between ln(sNfL) at admission and mRS scores (Table 4). After adjusting for age, sex, stroke history, and arrival time, the same results were found (Table 4).

## 4. Discussion

We found that age, sex, stroke history, and arrival time have crucial influences on sNfL levels at admission in patients suffering from AIS, despite the degree of the brain lesion, which is generally consistent with previous studies and our experience [16, 17]. The sNfL levels at admission were found to be positively correlated with the ASPECT scores and neurological deficits and negatively correlated with the prognosis of AIS patients. The enrolled patients were divided into revascularized and nonrevascularized groups based on treatment modality. We found that the NIHSS scores were higher, and the sNfL levels at admission were lower in the revascularized group than in the nonrevascularized group.

This interesting result may be related to the screening criteria for revascularization therapy [18]. Patients with revascularization had a higher rate of large vessel occlusion and greater ischaemic volume than patients who are not revascularized and exhibited higher NIHSS scores. However, the neurological deficits in the revascularized group were caused by the combination of core infarction and penumbra area [19, 20]. Ischaemia in the penumbra area may not rapidly cause destruction of neuronal cells and release of NfL into the blood. Another reason is that the arrival time was shorter in the revascularized group than in the nonrevascularized group in this study. The poststroke sNfL levels increased gradually over the extended course of disease [21]. Thus, the revascularized group had higher NIHSS scores but lower sNfL levels.

Previous work has indicated a positive correlation between sNfL levels and NIHSS scores in AIS patients [22]. In the present study, we found that sNfL levels at admission were positively correlated with NIHSS scores in AIS patients in the nonrevascularized group, consistent with previous studies. However, in patients who were successfully revascularized, we found that sNfL levels at admission were not correlated with NIHSS scores at either admission or 7 days poststroke. These results suggested that the role of sNfL at admission as a biomarker of AIS is influenced by revascularization therapy. Effective revascularization can rapidly improve the neurological function brought about by the penumbra area and reduce the NIHSS score of patients [23]. Therefore, we propose that sNfL levels at admission can reflect neurological deficits in AIS patients during the natural course of the disease, but this correlation is influenced by effective revascularization.

We assessed the neurological recovery and prognosis of patients by two indicators: the difference between the NIHSS score at admission and the NIHSS score 7 days after admission (NIHSS score difference) and the mRS score at 6 months after stroke. We found a negative correlation between the NIHSS score difference and sNfL levels in all patients and in the nonrevascularized patients, suggesting that higher sNfL levels predicted worse early neurological recovery. Regression analysis between mRS scores at 6 months after stroke and sNfL at admission suggested that sNfL at admission could be used to predict the long-term prognosis of nonrevascularized AIS patients. These results further suggest that effective revascularization can influence early neurological recovery and the long-term prognosis of AIS patients.

In conclusion, this study confirms that sNfL levels at admission can be used as a biomarker of AIS, but its predictive effect is mainly valid in patients who fail to receive revascularization therapy. When we consider sNfL as a clinical reference for the diagnosis and management of AIS, a high sNfL level at admission may imply more severe symptoms and worse prognosis. For patients with indications for endovascular therapy, patients with high sNfL levels at admission have a higher strength of recommendation for revascularization therapy.

Considering actual clinical situations, the time of patient arrival was used as the sampling node in this study. Although this measure will be confounded by the arrival time, this choice is more relevant to clinical application. Of course, it must be acknowledged that the arrival time is an important factor influencing the sNfL levels, which is a limitation of this study. Previous studies have found that sNfL levels begin to rise slowly at the beginning of the onset and peak at the end of 3 months after stroke [21]. For patients with nonmassive infarction, the rising trend was slow in the first week [24]. The present study confirmed that for AIS within 7 days, the arrival time does not significantly affect the correlation of sNfL with NIHSS scores and mRS scores. It can be anticipated that with more detailed research on sNfL trends and corrected data, sNfL is expected to be an important basis for guiding clinical treatment in the future.

## Figures and Tables

**Table 1:** Patient characteristics.

	Total	Revascularized	Nonrevascularized	*p* value
Demographic data				
Age, median (IQR) (y)	66.00 (18.25)	66.00 (18.00)	66.00 (18.00)	0.939
Female, *n* (%)	46 (40.70)	20 (46.50)	26 (37.10)	0.333

Laboratory values, median (IQR) (pg/mL)				
ln(sNfL)	3.39 (1.76)	3.08 (1.46)	3.81 (1.70)	0.014

Medical history, *n* (%)				
Heart disease	44 (38.90)	22 (51.20)	22 (34.10)	0.072
Hypertension	66 (58.40)	27 (62.80)	39 (55.70)	0.692
Diabetes	33 (29.20)	9 (20.90)	24 (34.30)	0.137
Smoke history	34 (30.10)	15 (34.90)	19 (27.10)	0.527
Drink history	33 (29.20)	18 (41.90)	15 (21.40)	0.034
Stroke history	23 (20.40)	10 (23.30)	13 (18.60)	0.636

Clinical data, median (IQR)				
ASPECT scores	12.00 (3.00)	10.00 (3.00)	13.00 (1.75)	0.001
NIHSS admission	8.00 (10.00)	14.00 (10.00)	6.00 (6.00)	0.005
NIHSS (7D)	4.00 (9.00)	3.00 (13.00)	3.00 (7.00)	0.162
Good prognosis, *n* (%)	78 (70.90)	28 (75.68)	50 (64.94)	0.165
Arrival time	7.00 (20.50)	4.00 (4.00)	15.00 (18.00)	<0.001

sNfL: serum neurofilament light chain; ASPECT: Alberta Stroke Program Early CT; NIHSS: National Institutes of Health Stroke Scale; mRS: modified Rankin Scale. Prognosis (good prognosis, mRS scores 0-2; poor prognosis, mRS scores 3-6).

**Table 2:** Determinants of sNfL levels within the study population.

	Unadjusted		Adjusted	
	*β* (SE)	*p* value	*β* (SE)	*p* value
Age	0.03 (0.01)	0.001	0.04 (0.01)	<0.001
Sex	0.69 (0.27)	0.013	0.64 (0.21)	0.003
Hypertension	-0.14 (0.22)	0.515		
Diabetes	0.35 (0.23)	0.138		
Smoke	-0.20 (0.31)	0.947		
Drink	0.15 (0.21)	0.639		
Stroke history	0.69 (0.27)	0.013	0.74 (0.25)	0.004
TIA history	0.62 (0.49)	0.207		
Binswanger's	-0.28 (0.24)	0.240		
LDL	-0.17 (0.14)	0.244		
HDL	0.51 (0.39)	0.197		
TG	0.14 (0.07)	0.059		
Δ time	0.01 (0.01)	0.050	0.01 (0.01)	0.042

sNfL: serum neurofilament light chain; TIA: transient ischaemic attacks; LDL: low-density lipoprotein; HDL: high-density lipoprotein; TG: triglyceride; Δ time: time between the onset of illness and arrival at the hospital.

**Table 3:** Associations between ln(sNfL) and clinical severity (NIHSS scores).

NIHSS admission	Total		Revascularized		Nonrevascularized	
	*p* value	*β* (95% CI)	*p* value	*β* (95% CI)	*p* value	*β* (95% CI)
Univariate for ln(sNfL)	0.875	0.015 (-0.182-0.212)	0.037	-0.346 (-0.590--0.079)	0.007	0.313 (0.096-0.506)
Multivariate for ln(sNfL)^∗^	0.293	0.019 (-0.020-0.0.050)	0.729	-0.013 (-0.080-0.060)	0.005	0.057 (0.019-0.060)

NIHSS (7D)						
Univariate for ln(sNfL)	0.003	0.279 (0.074-0.447)	0.568	-0.098 (-0.432-0.269)	<0.001	0.386 (0.188-0.547)
Multivariate for ln(sNfL)^∗^	0.004	0.041 (0.012-0.068)	0.967	0.002 (-0.064-0.077)	0.003	0.047 (0.019-0.085)

NIHSS (admission-7D)						
Univariate for ln(sNfL)	<0.001	-0.370 (-0.501--0.216)	0.080	0.023 (-0.548-0.084)	0.010	-0.299 (-0.475--0.083)
Multivariate for ln(sNfL)^∗^	0.017	-0.049 (-0.083--0.018)	0.606	-0.019 (-0.079-0.068)	0.083	-0.051 (-0.138-0.004)

^∗^Adjusted for age, sex, stroke history, and the time between the onset of illness and arrival at hospital. sNfL: serum neurofilament light chain; 7D: 7 days after stroke; NIHSS: National Institutes of Health Stroke Scale.

**Table 4:** Bivariate logistic regression analyses with mRS scores at 6 months as a dependent variable.

	ln(sNfL) of total			ln(sNfL) of revascularized			ln(sNfL) of nonrevascularized	
	*p* value	*β* (95% CI)		*p* value	*β* (95% CI)		*p* value	*β* (95% CI)
Model 1	0.338	1.216 (0.815-1.815)		0.120	0.435 (0.153-1.242)		0.039	1.762 (1.029-3.018)
Model 2	0.125	1.404 (0.910-2.165)		0.267	0.535 (0.177-1.615)		0.022	1.944 (1.101-3.431)
Model 3	0.065	1.549 (0.973-2.465)		0.224	0.493 (0.158-1.542)		0.011	2.208 (1.196-4.075)

CI: confidence interval; 6M: 6 months after stroke; mRS: modified Rankin Scale; Model 1: age and sex; Model 2: age, sex, and stroke history; Model 3: age, sex, stroke history, and the time between the onset of illness and arrival at the hospital.

## Data Availability

The data used to support the findings of this study are available from the corresponding authors upon request.
